# Laryngeal Fibrolipoma: A Case Report

**DOI:** 10.7759/cureus.76998

**Published:** 2025-01-06

**Authors:** Luis Ángel Garza-Montelongo, José Rosmal Cortés-Ponce, Nadia Gabriela Jasso-Ramírez, José Luis Trevino-González, Hayde Sarahí Ramos-Marrero

**Affiliations:** 1 Department of Otorhinolaryngology-Head and Neck Surgery, Hospital Universitario "Dr. José Eleuterio González", Monterrey, MEX; 2 Department of Pathological Anatomy and Cytopathology, Hospital Universitario "Dr. José Eleuterio González", Monterrey, MEX

**Keywords:** dysphonia, excision, fibrolipoma, laryngeal tumor, lipoma

## Abstract

Fibrolipoma is a benign mesenchymal tumor with a rare presentation in the aerodigestive tract. This lesion is characterized by a slow growth rate and symptoms such as dysphagia, dysphonia, and dyspnea due to the mass effect on neighboring structures. Achieving optimal surgical outcomes requires indirect laryngoscopy and imaging techniques such as computed tomography (CT) or magnetic resonance imaging (MRI), which provide useful information for a better understanding of the underlying pathology.

Surgery is the treatment of choice, whether using an external or endoscopic approach. Complete excision of the lesion is essential because of the elevated risk of local recurrence. This case report presents a patient diagnosed with laryngeal fibrolipoma who underwent an external approach to the lesion, highlighting the need for a post-surgical follow-up due to the substantial risk of recurrence, even after prolonged disease-free intervals.

## Introduction

Fibrolipoma is a rare type of tumor, representing only 1.6% of all facial lipomas; it can rarely affect the upper aerodigestive tract, accounting for 0.6% of all benign aerodigestive tumors [1]. These tumors can manifest as a pseudocystic or pedunculated mass, with no specific symptoms, highlighting the need for a correct differential diagnosis in patients with head and neck tumors. To date, no more than 100 cases of fibrolipoma have been reported [2]. In this case report, we present a laryngeal fibrolipoma case that presented as a pseudocystic mass, originating from the paraglotic fatty tissue. After surgical excision and histological testing, it was diagnosed as a lipoma composed of mature adipocytes.

## Case presentation

**Figure 1:**
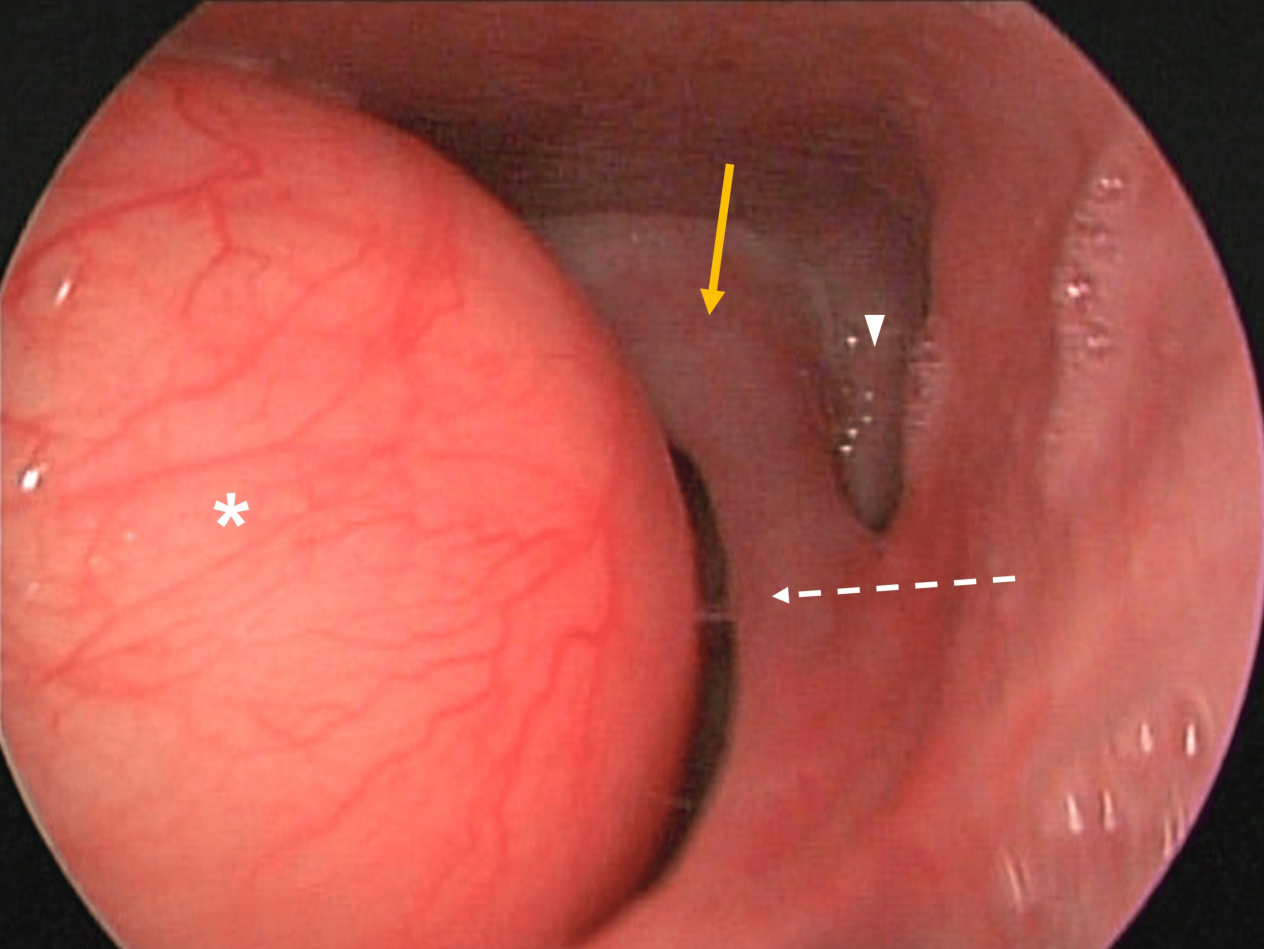
Laryngoscopic visualization of the tumor Image of fibrolaryngoscopy that shows a submucous, smooth, circumferential tumor in the hypopharynx (asterisk), dependent on the right pharyngeal wall, which obstructs the visual field (yellow arrow: left arytenoid; white dotted arrow: left aryepiglottic fold; arrowhead: left pyriform sinus).

We present the case of a 51-year-old male patient who attended the Otolaryngology-Head and Neck Surgery Division of the University Hospital “Dr. José Eleuterio González,” a tertiary care referral center in northeast Mexico, referring a two-year-onset dysphonia associated with six months of progressive dyspnea and orthopnea. Indirect laryngoscopy was performed, showing an exophytic, pinkish, regular, circumferential supraglottic lesion, which obstructed the hypopharynx (Figure 1).

**Figure 2:**
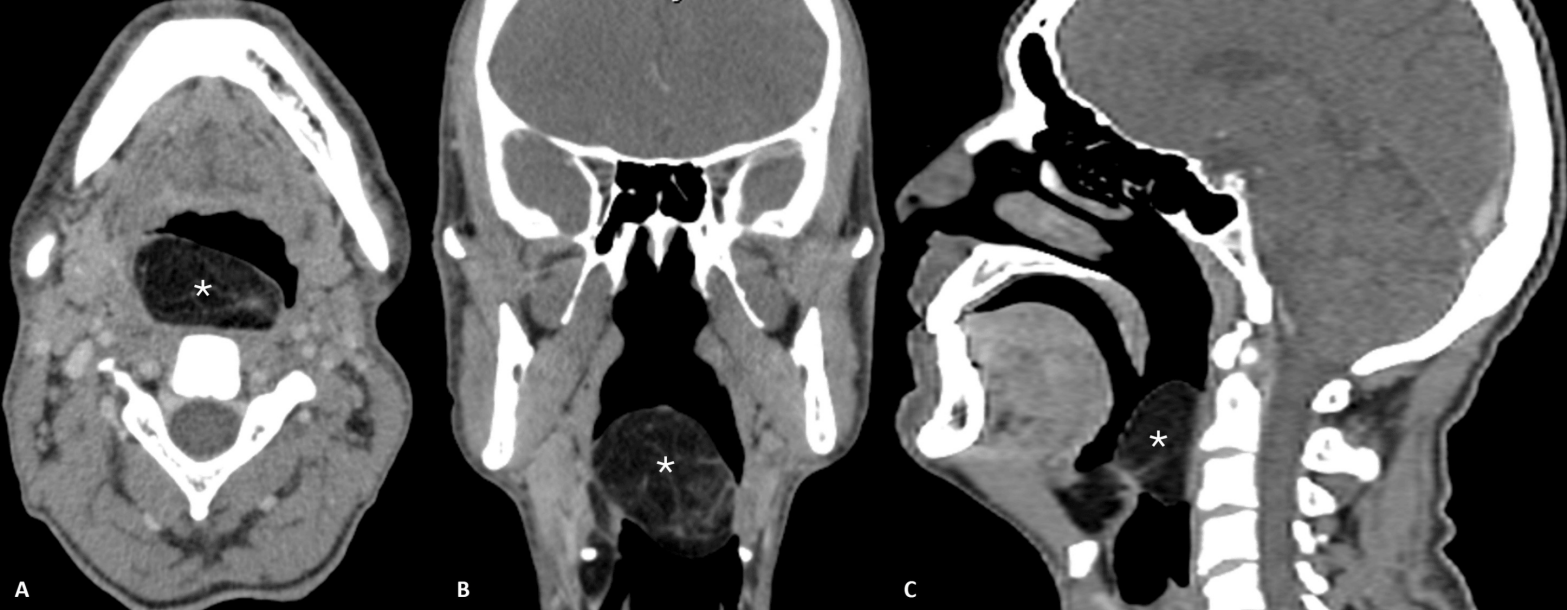
Contrast CT scan Computed tomography in transversal (A), coronal (B), and sagittal (C) views, performed at the time of diagnosis, demonstrates a low-density lesion with an apparent origin in the paraglotic space, which crosses the midline and extends to the oropharynx, obstructing the upper aerodigestive tract (asterisk).

Given upper airway obstruction, the patient was admitted to the emergency department, where venous blood gas parameters and computed tomography (CT) were conducted (Figure 2). The CT revealed a submucosal lesion at the right paraglotic fatty tissue, which ascended and protruded through the thyrohyoid membrane and extended to the ipsilateral vallecula, epiglottis, and posterior pharyngeal wall. The lesion measured 62 x 30 x 51 mm, obstructing 73% of the hypopharynx, with characteristics suggestive of fibrolipoma. The rest of the studies performed showed no abnormalities. An urgent tracheostomy was performed under local anesthesia, and no complications were identified. Once the upper airway was secured, a biopsy guided by microlaryngoscopy was performed, reporting a histological diagnosis of fibrolipoma.

**Figure 3:**
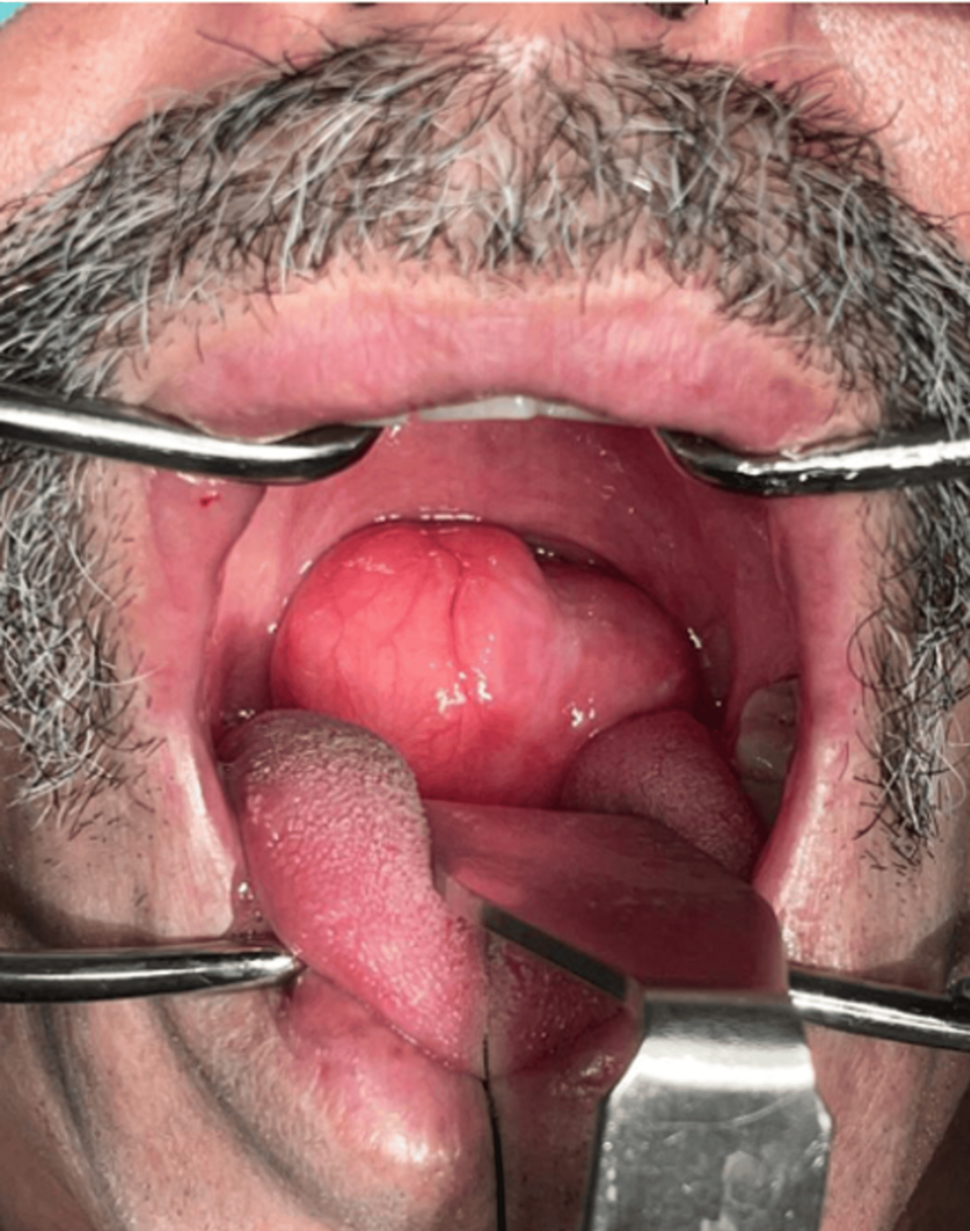
Intraoperative oral examination Intraoperative oral examination revealed tumor adhesion fibers to the right tonsil. Laryngosuspension and microscopic surgery were attempted, but due to aerodigestive obstruction, they were abandoned.

Throughout the inpatient care, the subject was diagnosed with pulmonary tuberculosis and referred to the pneumology department for treatment. By the time the therapeutic regimen was completed, the tumor excision was performed using a combined technique. During the preoperative assessment, an increase in the volume of the tumor was identified; the lesion was now visible through the oropharynx, in contact with the hard palate, and showed adhesion fibers with the right tonsil (Figure 3).

**Figure 4:**
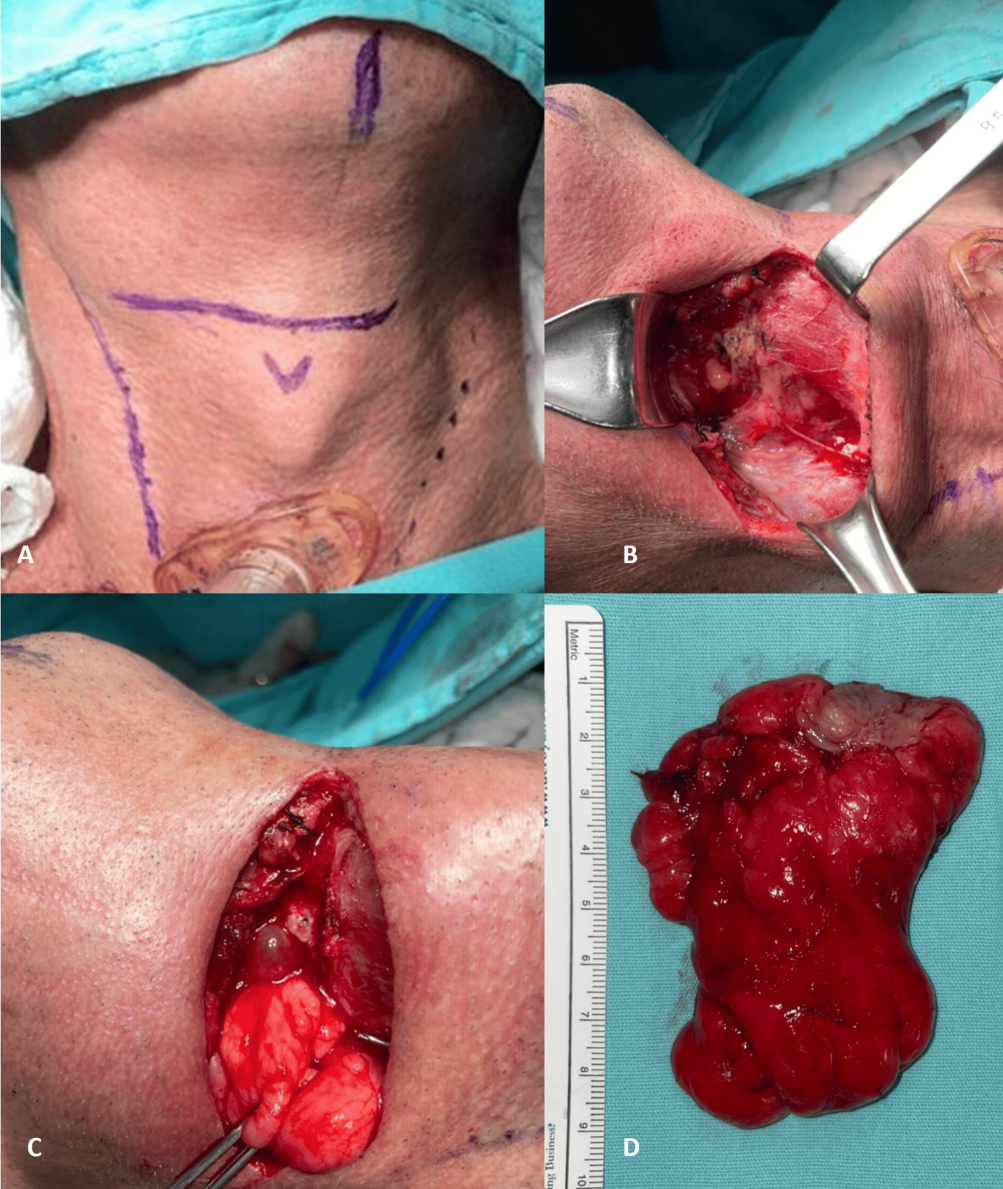
Surgical approach and specimen A-C: External surgical approach with an almost immediate demonstration of tumor herniation; D: Surgical specimen

During the surgical procedure, the removal of the adhesion fibers was performed using a bipolar electrocautery. The tumor excision began with a 15 cm right cervicotomy and dissection of the platysma muscle, localizing the hyoid and dissecting the suprahyoid muscle. A lateral pharyngotomy was conducted afterward, resecting the entire lesion through it. A fibrous stalk-shaped origin of the lesion was identified on the anterior pharyngeal wall (Figures 4A-4C). A nasogastric tube was placed, and the surgical approach was closed, leaving a Penrose drain in place. Subsequently, a new laryngoscopy was conducted to look for anomalies in the pharyngeal wall. The final histopathological examination revealed a 140 g nodular tumor, measuring 5 x 4.5 x 4.5 cm, covered by a smooth and translucent capsule (Figure 4D).

**Figure 5:**
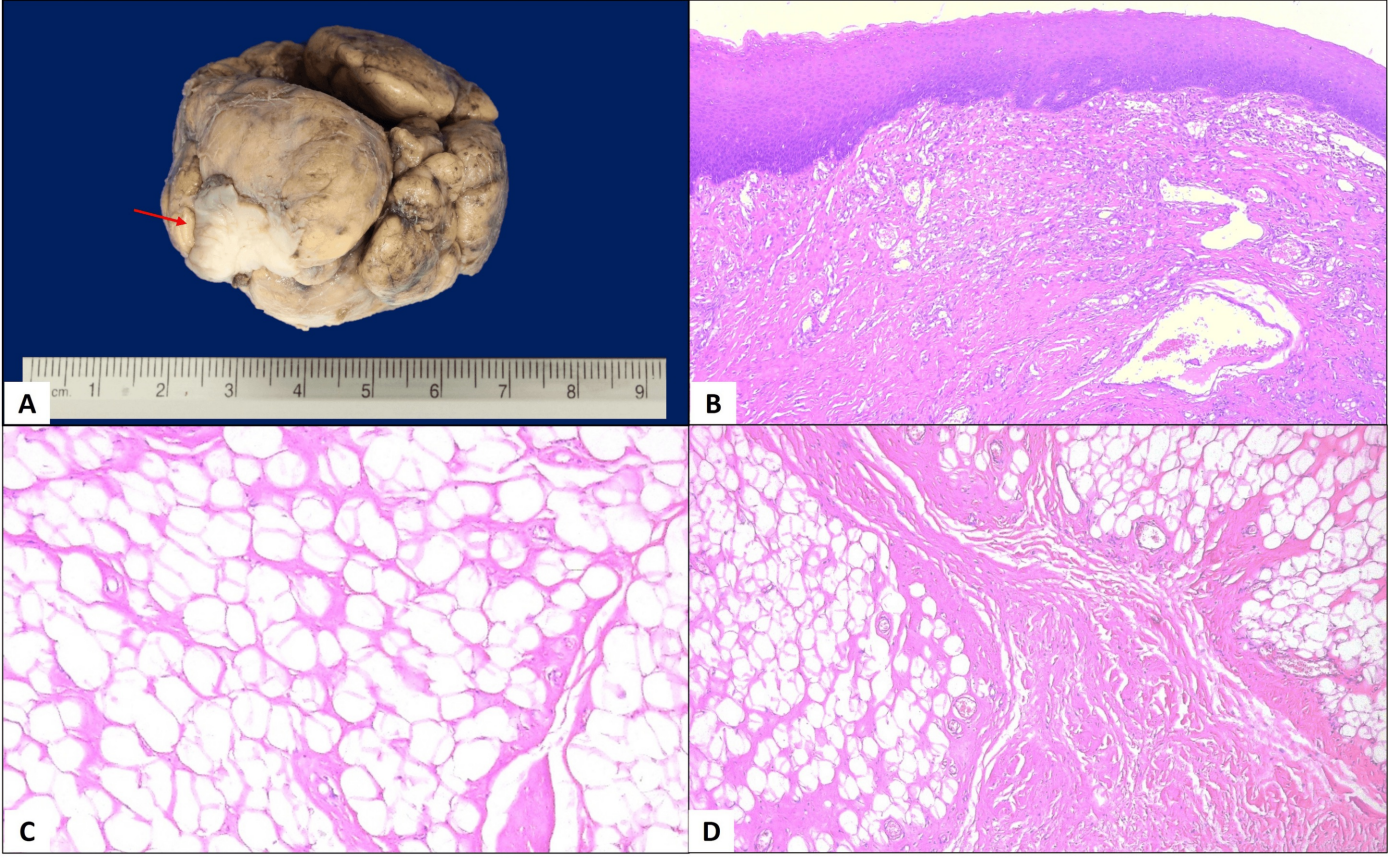
Histopathological characteristics of the surgical specimen A: Macroscopic image of the specimen, which weighed 140 g and measured 5 cm at its largest dimension. It was lobulated and yellowish in color. A small fragment of mucosa was observed (arrow); B: The mucosal fragment showed a proliferation of capillary vessels in the lamina propria and a mild chronic inflammatory infiltrate (H&E, 5X); C: he specimen was composed of mature adipose tissue (H&E, 10X); D: Certain areas of the tissue displayed prominent fibrosis (H&E, 5X).

The cross-section cut of the tumor showed a lobulated and homogeneous solid adipose content, with numerous congested capillaries and a central 4 cm fibrous tract, consistent with fibrolipoma (Figure 5).

The patient underwent the postoperative period without complications and was discharged on the fifth postoperative day. Follow-up involved performing an indirect laryngoscopy 11 days after the surgery; no anomalies were detected, and both the nasogastric tube and Penrose drain were removed. An oral diet was initiated, and the patient was scheduled for monthly follow-up visits, which were favorable and without complications. In the third postoperative month, tracheostomy decannulation was performed. Imaging studies at six months and one year showed no recurrence of the tumor.

## Discussion

First described by Roux in 1848 and designated as “yellow epulis” [3], lipoma is a benign mesenchymal tumor composed of mature adipocytes. It has been reported that 15%-20% of lipoma cases involve the head and neck region, while 1-4% affect the oral cavity. The WHO classifies lipomas into conventional lipoma, fibrolipoma, angiolipoma, nerve lipomatosis, and lipoblastoma [4]. There are only a few histological subtypes of fibrolipoma, unlike conventional lipoma. Lipomas are benign mesenchymal tumors with a slow growth rate, representing 4%-5% of all benign tumors in humans [5]. Lipomas are more commonly found on the trunk and limbs, where subcutaneous cellular tissue is abundant. In the case of large tumors, clinical manifestations can involve aesthetic concerns and may affect tissue function as a result of their anatomical location. It is estimated that 13%-15% of lipomas can affect the head and neck region, while upper airway involvement is exceedingly rare [6]. Lipomas represent a dysplasia of slow growth, circumferential in nature, with a smooth fibrous capsule. They can present as a single lesion or multiple lesions as a clinical manifestation of conditions such as neurofibromatosis, Gardner syndrome, Launois-Bensaude syndrome, Madelung disease, or Dercum disease [7].

Many authors have proposed that lipomas arise from embryogenic lipoblastic cells or metaplastic muscular cells, with a probable familial and endocrine etiopathogenic background [8,9]. Traumas, infections, and chronic irritative conditions have also been associated. A study by Murty et al. reported that lipomas could develop as a result of fibroblasts being multipotential cells that can differentiate into fatty cells, leading to lipoma formation [10]. To date, no specific risk factors for lipomas have been identified [11].

It has been reported that 28% of lipomas develop after the fifth decade of life, and 27% of these tumors develop between the third and fourth decades of life. Lipomas are more frequently diagnosed in males (62.5%) [12]. There are different subtypes of lipomas namely, mixolipoma, fibrolipoma, fusiform cell lipoma, angiolipoma, and pleomorphic lipoma, which are the most commonly diagnosed [13]. The possibility of malignant transformation in a lipoma is extremely infrequent but increases in cases of multiple laryngopharyngeal lipomatosis [14,15].

It is well known that laryngeal lipomas are extremely uncommon. When they do occur, laryngeal lipomas affect the supraglottic larynx and develop from the fat tissue of the laryngeal vestibule, aryepiglottic fold, and epiglottis [15]. Owing to their slow growth rate, lipomas are typically asymptomatic for a long period and measure 5-6 cm, but once they become large, they cause symptoms as a consequence of anatomical compression. Lipomas of the upper aerodigestive tract can manifest symptoms such as dysphagia, dysphonia, a sensation of pharyngeal globus, cough, and, in cases of airway obstruction, stridor, and dyspnea, which may lead to asphyxia and cardiorespiratory arrest [16], leading to the need of an urgent tracheostomy, as was the case presented in this report.

During endoscopic evaluation with fiberoptic flexible laryngoscopy, a lipoma may appear as a submucosal or polypoid mass, and it can sometimes be pedunculated. It is recommended to always perform a biopsy if possible [17]. Preoperative diagnosis can be achieved through imaging techniques such as CT or magnetic resonance imaging (MRI). On CT, lipomas appear as homogeneous lesions with low attenuation values of ionizing radiation (0 Hounsfield units), and their density is lower than water. A CT scan can accurately determine the tumor’s extent and has a diagnostic precision rate of 75%-90%. On the other hand, MRI provides better soft tissue definition, making it the preferred imaging technique [18].

Surgery is the elective treatment for laryngeal lipomas. It is preferred to perform a conservative endoscopic procedure in the case of a small tumor [18]. In contrast, larger tumors (> 2 cm), submucosal tumors, or those that may be malignant require an external surgical approach, such as lateral pharyngotomy, laryngofissure, or subhyoid pharyngotomy [19]. Surgical excision of the lipoma needs to be performed to avoid recurrence [20].

Postoperative care includes, based on our experience, nutrition through a nasogastric tube for a few days. After deglutition rehabilitation, the oral diet can be initiated.

## Conclusions

The laryngopharyngeal fibrolipoma is a rare tumor that exhibits slow growth and may represent a significant risk of upper airway obstruction due to its anatomical location. Although uncommon, a lipoma should be considered one of the differential diagnoses of midline laryngeal masses. Computed tomography and MRI are the primary imaging modalities used to characterize these lesions, establish the tumor's origin, and delineate the extent of the disease. Furthermore, complete excision of the lesion is essential to prevent recurrence.

## References

[1] González-Olivares H, Juárez-Rebollar AG, Siordia-Reyes AG (2019). Lipoma of the maxillofacial region in pediatric patients. A case study. Rev Odont Mex.

[2] De Vincentiis M, Greco A, Mascelli A, Soldo P, Zambetti G (2010). Lipoma of the larynx: a case report. Acta Otorhinolaryngol Ital.

[3] Verma N, Srivastava P (2015). Lipoma in soft palate: a rare presentation. J Res Adv Dent.

[4] (2024). Categoría D17 sobre lipomas (Website in Spanish). https://www.paho.org/es/relacsis/foro-dr-roberto-becker/categoria-d17-sobre-lipomas.

[5] Cawson RA (1995). Soft tissue tumors, 3rd edition, 1995: F.M. Enzinger and S.W. Weiss. Mosby, St Louis, £160. Eur J Cancer B Oral Oncol.

[6] Jungehülsing M, Fischbach R, Pototschnig C, Eckel HE, Damm M (2000). Rare benign tumors: laryngeal and hypopharyngeal lipomata. Ann Otol Rhinol Laryngol.

[7] Persaud RA, Kotnis R, Ong CC, Bowdler DA (2002). A rare case of a pedunculated lipoma in the pharynx. Emerg Med J.

[8] Trizna Z, Forrai G, Toth B, Banhidy FG (1991). Laryngeal lipoma. Ear Nose Throat J.

[9] Schuler FA 3rd, Graham JK, Horton CE (1976). Benign symmetrical lipomatosis (Madelung's disease). Case report. Plast Reconstr Surg.

[10] Murty KD, Murty PS, George S, Balakrishnan R, Mathew KJ, Varghese G (1994). Lipoma of the larynx. Am J Otolaryngol.

[11] Enzi G (1984). Multiple symmetric lipomatosis: an updated clinical report. Medicine (Baltimore).

[12] Singhal SK, Virk RS, Mohan H, Palta S, Dass A (2005). Myxolipoma of the epiglottis in an adult: a case report. Ear Nose Throat J.

[13] El-Monem MH, Gaafar AH, Magdy EA (2006). Lipomas of the head and neck: presentation variability and diagnostic work-up. J Laryngol Otol.

[14] Wenig BM (1995). Lipomas of the larynx and hypopharynx: a review of the literature with the addition of three new cases. J Laryngol Otol.

[15] Laurent C, Lindholm CE, Nordlinder H (1985). Benign pedunculated tumours of the hypopharynx. 3 case reports, 1 with late malignant transformation. ORL J Otorhinolaryngol Relat Spec.

[16] Barry B, Charlier JB, Ameline E, Nallet E, Depondt J, Géhanno P (2000). Retro-pharyngeal and pharyngeal-laryngeal lipomas (Article in French). Ann Otolaryngol Chir Cervicofac.

[17] Tien RD, Hesselink JR, Chu PK, Szumowski J (1991). Improved detection and delineation of head and neck lesions with fat suppression spin-echo MR imaging. AJNR Am J Neuroradiol.

[18] Sharudin SN, Thangavelu T, Roslim SN, Hitam S, Mat Baki M (2022). Large vallecula epiglottica lipoma: a rare but fatal cause of dysphagia. Cureus.

[19] Durr ML, Agrawal N, Saunders JR, Ha PK (2010). Laryngeal lipoma associated with diffuse lipomatosis: case report and literature review. Ear Nose Throat J.

[20] Iannella G, De Vincentiis M, Corsi A, Greco A, Magliulo G (2017). A rare case of embryonal rhabdomyosarcoma of the parapharyngeal space. Acta Otorhinolaryngol Ital.

